# Material Disadvantage and Positive Subjective Health of Older Homeless and Older Irish Travelers

**DOI:** 10.1093/geroni/igaa057.2510

**Published:** 2020-12-16

**Authors:** Bridin Carroll, Kieran Walsh

**Affiliations:** 1 NUI Galway, Galway, Galway, Ireland; 2 Irish Centre for Social Gerontology, National University of Ireland Galway, Galway, Galway, Ireland

## Abstract

Focusing on older Irish Travellers and older homeless people (OTOH) as two marginalised sub-sections of the older population, this paper investigates life-course and structural forms of material disadvantage, and its implications for positive health and accessing community care in older age. With growing interest in strengthening home care structures for older people, it is critical to interrogate the relevance of these structures for those who experience environmental uncertainty in later life, and possess significant trajectories of disadvantage. The analysis draws on 50 life-course interviews with OTOH aged between 50-72 years. The findings illustrate significant life-course experiences of material and multi-faceted forms of disadvantage, including stigma and discrimination, with implications across health and social lives. Housing deprivation was a multi-factorial player, causing certain physical illnesses, hindering some health treatments, and contributing to precarious conditions and sense of self worth. Findings are discussed in relation to flexible models of home care delivery.

